# Lightweight design based on automotive drive axle housing

**DOI:** 10.1371/journal.pone.0331300

**Published:** 2025-09-18

**Authors:** Yingshuai Liu, Chenxing Liu, Jianwei Tan, Yunli He

**Affiliations:** 1 School of Mechanical Engineering, Shandong Huayu University of Technology, Dezhou, China; 2 National Lab of Auto Performance and Emission Test, School of Mechanical and Vehicular Engineering, Beijing Institute of Technology, Beijing, China; TU Dublin Blanchardstown Campus: Technological University Dublin - Blanchardstown Campus, IRELAND

## Abstract

To address the limitations in the cruising range and improve the overall efficiency of Pure Electric Vehicles (PEVs), this study focuses on the lightweight design of PEV drive axles. Aligning with the current trend toward lightweight and integrated development in electric vehicles, the research is based on the concept of an integrated electric drive axle housing. A three-dimensional model of the electric vehicle axle was developed using three-dimensional modeling software(3D modeling software), taking into account the working principles and load characteristics of the electric drive axle. Subsequently, Finite Element Analysis (FEA) was performed using finite element analysis software to evaluate the stiffness, strength, and modal characteristics of the integrated electric drive axle housing. The study analyzed stress distribution, deformation patterns, and natural frequency ranges under various operating conditions. Based on the FEA results, the axle housing structure was optimized by reducing the wall thickness and modifying the material of components subjected to lower stress levels, with the goal of minimizing mass. The optimal solution involved adjusting the housing thickness to achieve a more uniform and efficient thickness distribution, thereby meeting the lightweight design objectives. Simulation verification of the optimized axle structure confirmed that the weight reduction was achieved without compromising the required strength and stiffness. The findings demonstrate that, while ensuring safety, the optimized axle structure achieved a 12% reduction in weight. This study provides a feasible solution and a solid theoretical foundation for advancing the development of lightweight electric vehicles.

## Introduction

With the increasingly serious global energy crisis and environmental pollution, the development of pure electric vehicles has gradually become a solution to alleviate the energy crisis. As an emerging clean energy vehicle, the market demand for pure electric vehicles has been growing, and the market ownership rate has reached a record high [1]. But the popularity and efficiency of electric vehicles are mainly limited by the battery range [2]. Among them, the design optimization of the drive axle, as one of the important load-bearing components of the vehicle, is very critical. Therefore, exploring the lightweight design of drive axles for EVs can not only reduce the total vehicle weight and extend the range, but also improve the overall performance and energy efficiency of the vehicle.

The power consumption of a vehicle is directly proportional to its weight, i.e., the greater the total weight of the vehicle, the higher its power consumption. According to research data, a 10 per cent reduction in the overall vehicle weight of a vehicle can increase the power conversion efficiency by 6–8 per cent [3]. In addition, the acceleration performance of a car is closely related to its mass. According to the formula for calculating the kinetic energy of a car, the larger the mass of the car, the greater the energy required. Axle weight reduction means a reduction in the overall mass of the car, which will shorten the acceleration time of the car and improve the starting performance of the car. For example, if the total mass of a car is reduced from 1,500 kg to 1,482 kg [4], although the reduction is not significant, this weight reduction effect can still significantly improve the power performance of the car when driving at high speeds or under frequent acceleration. In addition, reducing the weight of the axle also improves the braking performance of the car. A lighter axle means less inertia for the braking system to overcome, allowing for faster deceleration and stopping. This is important for improving driving safety and reducing braking distances [5].

Therefore, researchers are actively working on the lightweight design of automotive axles and have achieved impressive results by using different innovative methods. Guo D. [6] conducted Multi-Body Dynamics (MBD) and Finite Element Analysis (FEA) on the drive axle housings of off-road vehicles, and evaluated the stress distribution, deformation, and fatigue damage of the drive axle housings by simulating the loads under different operating conditions, which significantly improved the performance of the drive axle housings under complex road conditions. The performance and life of the axle housing under complex road conditions were evaluated by simulating the loads under different conditions, which significantly improved the performance and life of the axle housing under complex road conditions, but no further model construction was carried out; Zhou S [7] carried out dynamic response and fatigue analyses of the axle housing of the drive truck of an agricultural vehicle, and experimentally verified the accuracy of the results, and proposed a method for optimal design, but did not carry out the analysis of the data of the FEA. Stabile P et al. [8] used high-strength steel and carbon fiber composite materials for optimal design, and successfully reduced the fatigue damage of the drive axle housing by using the MBD and FEA composites for optimal design, successfully reducing the mass of the drive axle housing by 12%. The successful application of this design gives full play to the advantages of the materials and improves the strength and rigidity of the axle. Jin D et al. [9]optimized the drive axle structure using a multi-body integrated design approach and successfully achieved a 15% reduction in axle mass. This approach focuses on the synergy of the whole drive axle system and provides an effective way to improve the performance of the whole vehicle. Johnson M.S. [10] successfully reduced the mass of the axle housing by 20% through digital manufacturing technology, which is a significant progress in the manufacturing process. However, these studies generally did not propose subsequent solutions.

The application of digital manufacturing technology provides higher precision and flexibility for lightweight design and promotes technological innovation in pure electric vehicles. In this study, an integrated drive axle housing is explicitly proposed as a research focus from the perspective of lightweighting axles for pure electric vehicles. The reliability study of the integrated drive axle housing for pure electric vehicles is carried out by establishing a three-dimensional model combined with finite element analysis. The main research content is:

(1) By analyzing and summarizing the relevant research results at home and abroad, the composition, operation mechanism and practical application environment of the axle of pure electric vehicles are deeply grasped. The specifications of key components, such as the motor and transmission reducer, are designed in detail to ensure that their theoretical performance meets the basic operating requirements of electric vehicles. It lays a solid theoretical and practical foundation for the subsequent strong transition and stiffness characteristic analysis, modal frequency, as well as lightweight design and fatigue life analysis.
(2) Firstly, the solid model of integrated axle is established by 3D modeling software according to the data, and the model is simplified according to the actual force condition of integrated axle shell and imported into finite element analysis software. Then analyses the stresses and displacements under the most extreme straight force condition, extreme driving force condition, emergency braking condition and extreme lateral force condition, and carry out a reasonable optimization design by analyzing the stress cloud diagram.
(3) Conduct a finite element modal analysis of the integrated drive axle in a pure electric vehicle. Identify and subsequently enhance the modal characteristics of the critical structural components within the axle housing to mitigate resonance phenomena during practical applications, ultimately improving the vehicle’s dynamic stability.
(4) According to the static analysis and modal analysis, the integrated drive axle housing of pure electric vehicle is optimized, and selected static modal analysis is performed again until it meets the design requirements and achieves the purpose of lightweighting.
(5) Based on the above research content, the specific results of lightweight design and the impact on the performance of electric vehicles are analyzed. A new research direction and an outlook on the lightweight design of axle housings for future electric vehicles are proposed.

## Design and creation of integrated drive axle housing models

### Integrated drive axle housing design description

The design is grounded in the principle of minimizing both manufacturing costs and the weight of the axle housing, while ensuring adequate structural integrity. This approach aims to enhance the driving range of pure electric vehicles (PEVs). Lightweighting not only contributes to reduced energy consumption but also enhances vehicle dynamics and handling performance. The primary design objective is to achieve maximum weight reduction in the axle without compromising safety and performance standards [11,12].

In the axle design, a symmetrical structure is employed to ensure balanced force distribution and torque output stability. The axle housing features an 8 mm wall thickness, with the half-shaft casing designed to an inner diameter of 90 mm and an outer diameter of 100 mm, ensuring optimal compatibility with bearings and rotating components. A wheelbase of 1520 mm is selected to meet vehicle stability requirements, while the spring seat spacing is set at 1000 mm to maintain the efficacy of the suspension system [13].

### Integrated drive axle housing modeling

**Fig 1:**
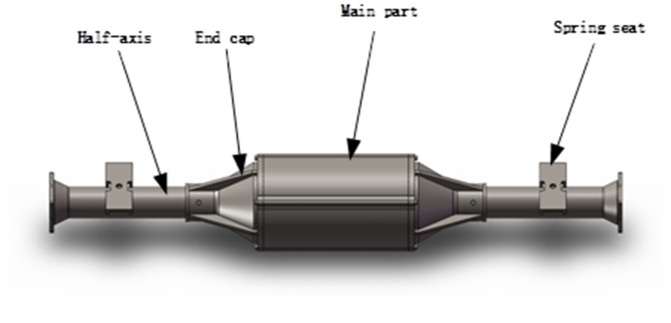
Integrated drive axle housing 3D model.

The 3D model of the axle housing is drawn by 3D modeling software modelling software [14–17]. The integrated drive axle housing is divided into three main parts: the body of the axle housing, the end caps on both sides, and the bushings for the half shafts. Considering the complex structure of the axle housing, it is necessary to use 3D modeling software to build its solid model, based on the specific dimensional parameters of each component, the use of 3D software to design the axle housing body, end cover and half shaft casing model. In order to ensure that the results are accurate and efficient, the model is simplified without affecting the mechanical properties of the axle housing, omitting the complex structural parts that are subjected to small or no force and the non-load-bearing attachments on the half-axle casing. Next, by realizing the assembly relationship between these parts, we will be able to assemble a complete 3D model of the direct drive axle. The 3D model of the housing is shown in Fig 1.

### Integrated drive axle housing data

40Cr steel was selected as the material for the axle housing, and the properties of 40Cr steel after tempering were determined through data enquiry, and the specific properties are shown in Table 1 [18] as follows.

**Table 1:** Material data for integrated drive axle housing.

Name	Modulus of elasticity/MPa	Poisson’s ratio	Yield strength/MPa	Material density/(kg/m^3^)
40Cr	208000	0.3	785	7845

The main parameters of an existing pure electric vehicle are 1.3t full axle load, 1610 mm distance between the wheels on both sides, 1320 mm distance between the two shock absorbing leaf springs, the detailed main parameters are shown in Table 2.

**Table 2:** Main parameters of the whole vehicle.

Name	Parameter	Name	Parameter
Wheelbase	1520mm	Full shaft load	1500 kg
Wheel radius	0.283m	Transmission ratio	6.5
Full axle load	1300 kg	Transmission efficiency	0.9
Motor reducer weight	97 kg	Peak motor torque	200Nm

### Integrated drive axle housing finite element simulation analysis

The drive axle, as a key structure supporting the weight of the vehicle body and as a core part of the drive train, experiences variable and complex operating conditions during the driving process of a pure electric vehicle [19–21]. Under these conditions the axle will be affected by vibration and noise, and if its strength and stiffness do not meet the standards it may be damaged, potentially increasing the risk of traffic accidents. Therefore, it is crucial to assess the strength and stiffness of drive axles under various extreme operating conditions.

### Principle of finite element analysis.

Finite Element Analysis (FEA) was employed to predict structural responses under external loads [22–25], which in turn predicts the physical response of a complex structure under external forces.

### Simplification of finite element model of integrated drive axle housing

**Fig 2:**
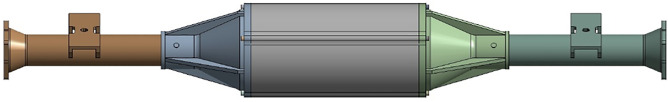
Finite element model of integrated drive axle housing.

In order to ensure that the analysis accuracy can meet the design requirements while improving the optimization calculation efficiency, it is usually necessary to simplify the model appropriately. On the premise of not affecting the core accuracy of the analysis results, those details that have less influence on the results are removed [26–29]. For example, for a complex structure such as an axle, we can omit those small features that do not bear stresses, such as threads, small holes, slender edges, etc., because these detail parts do not have much influence on the overall stress distribution, and reduce the amount of computation by simplifying the model. As shown in Fig 2.

### Integrated drive axle shell model meshing

**Fig 3:**
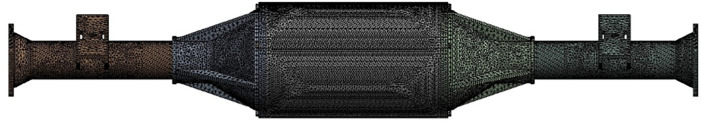
Integrated drive axle housing meshing.

The simplified model was imported into finite element analysis software for structural analysis. Mesh sensitivity was ensured by refining critical regions (5 mm) while coarsening low-stress areas (8.5 mm), resulting in 458,942 elements and 287,601 nodes (Fig 3). These tools provide a scientific computational basis and visualization of results for design optimization, product development, manufacturing processes and post-testing in the engineering field.

To ensure the reliability of finite element results, a mesh sensitivity study was conducted. Three mesh sizes were tested: Coarse (8.5 mm), Baseline (5 mm for critical regions), and Fine (3 mm). The maximum stress and displacement under the Maximum Vertical Load Condition were compared across these meshes (Table X). Results showed that the variation in stress between Baseline and Fine meshes was less than 2%, confirming mesh convergence. The selected baseline mesh (458,942 elements) was therefore deemed sufficient for accuracy while maintaining computational efficiency

To verify the reliability and convergence of the mesh size, a mesh sensitivity analysis was performed prior to formal simulation. The analysis aimed to determine the optimal mesh density that balances calculation accuracy and efficiency, ensuring that the results do not vary significantly with further mesh refinement. The simplified finite element model is imported into the finite element analysis software. Based on the mesh sensitivity results, appropriate mesh cell density is set to meet the accuracy requirements in different regions: in the region of high stress concentration or complex detail surfaces, a finer 5 mm mesh size is used; in areas of gentle stress distribution, a coarser 8.5 mm mesh is adopted. This method greatly increases the calculation speed while ensuring the quality of the calculation.

The final result is 458942 grid cells and 287601 nodes (as shown in Fig 3). The selection of 5 mm and 8.5 mm mesh sizes is based on the mesh sensitivity analysis: when the mesh size is 5 mm, the results are sufficiently converged (variation <0.5% compared to 3 mm), and increasing mesh density further (to 3 mm) only increases computational cost without significant improvement in accuracy. For low-stress areas, 8.5 mm is chosen as the results remain within acceptable error margins (<2% compared to 5 mm). Fig. 3 Integrated drive axle housing meshing.

### Integrated drive axle shell working condition analysis

In the face of the complexity and variability of the driving environment, the motion state of the vehicle is also changing all the time, which leads to the complexity of the vehicle stress situation. We can evaluate the performance of EVs by analyzing the following four critical working conditions: maximum vertical load condition, maximum traction load condition, emergency stop stress condition and extreme lateral force condition [30–34]. Once it is confirmed that the strength and stiffness of the drive rear axle housing of the EV meets the predefined criteria for use under these extreme conditions, we can conclude that the drive axle is suitable for EV use under real road conditions.

#### Maximum vertical load working condition.

When a fully loaded pure electric vehicle is travelling on an uneven road surface and ignoring the lateral forces it is subjected to impact loads under extreme conditions, the axle has to cope with the vehicle’s own fully loaded gravitational force G and the gravity of the electric drive system G1 as well as additional impacts from the unevenness of the road surface.

Impact load at the plate spring seat under maximum vertical force:


$$F_{L}=k_{d}\times G=k_{d}\times m\times g$$ (1)

Total force at the axle leaf spring seat:


$$F=\frac{G+F_{L}}{2}$$ (2)

The motor and reducer receive shock loads:


$$F_{M}=k_{d}\times G_{1}=k_{d}\times m_{1}\times g$$ (3)

Total force on the integrated bridge shell:


$$F_{2}=G_{1}+F_{M}$$ (4)

**Fig 4:**
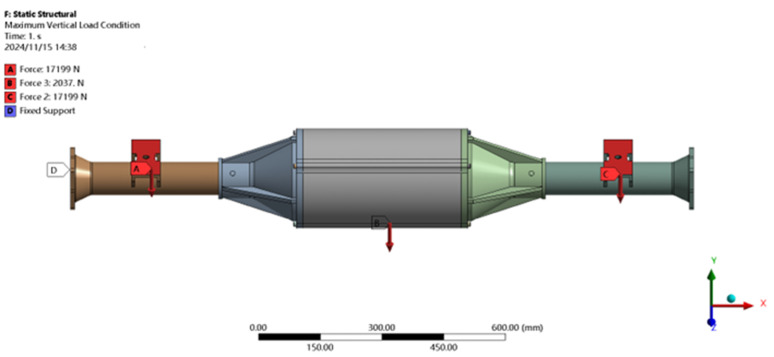
Constraint and load application at maximum vertical load.

In the formula, F is the total force at the steel plate spring seat; $F_{2}$ is the total force at the integrated axle shell; FL is the impact load at the steel plate spring seat; $F_{M}$ motor at the reducer is subjected to impact load; for the full load mass of the integrated axle of the electric vehicle is 1300 kg, for the mass of the car at the reducer is 77 kg, for the gravity acceleration is taken as the value of 9.8, and for the value of the dynamic load coefficient is taken as the value of 1.7;

Set load constraints: constraints integrated drive axle shell ends of X, Y, Z, direction of movement and X, Y direction of rotation, so that the end point can not occur displacement and turn to. As shown in Fig. 4.

Setting the loading load: after bringing the parameters into the formulae for calculation, it is learned that in this particular case, a vertically downward force of 17,199N is to be applied in the positive direction of the Y-axis of the spring steel plate seat, and a force of 2037N is also to be applied at the center of gravity of the bridge shell. As shown in Fig. 4.

**Fig 5:**
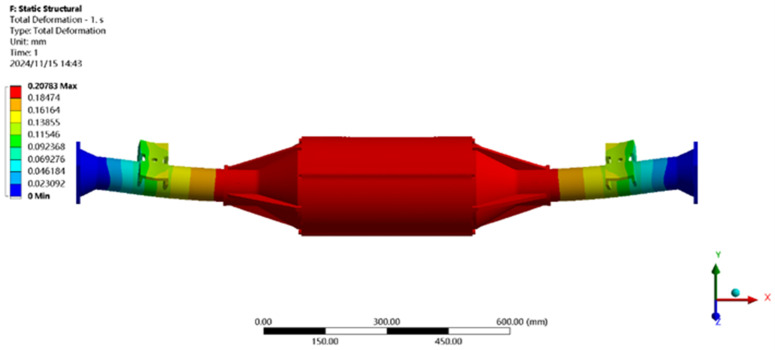
Maximum vertical load displacement clouds.

**Fig 6:**
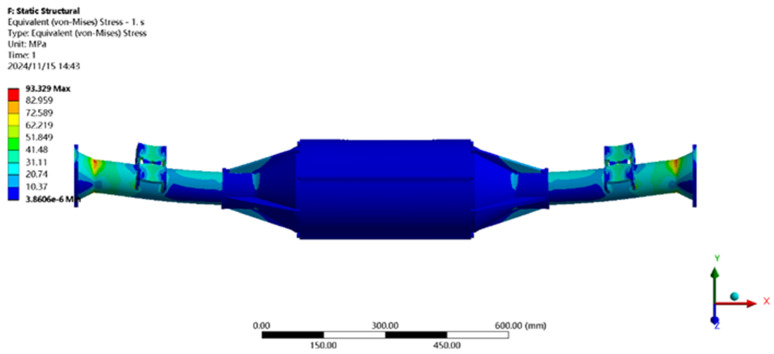
Stress cloud of maximum vertical loading.

The finite element analysis software is used to solve for the maximum vertical working condition, and the results of the integrated drive axle shell finite element simulation and analysis of the stress and displacement cloud are obtained as shown in Figs 5 and 6.

From the analysis results of the maximum vertical load condition displacement cloud diagram in Fig 5, it can be seen that the integrated drive axle shell displacement deformation gradually increases from both ends to the middle of the shell. The maximum deformation position in the bridge shell body motor and reducer position, the maximum deformation of about 0.208 mm, the variable per meter wheelbase is 0.208/1.520 ≈ 0.14 mm/m, much smaller than the national regulations of 1.5 mm/m, which is determined to meet the steel strength.

From Fig 6 maximum vertical load condition stress cloud analysis results can be seen integrated drive axle shell maximum stress is located in the half shaft casing at both ends of the constraints, where the maximum stress position of the von-Mises stress of 93.36MPa, less than the integrated drive axle shell material 40Cr yield strength of 785MPa. in other areas of the stress value is lower, are in line with the requirements of the performance specifications of the EV drive axle, which determines that the drive axle meets the strength requirements.

#### Maximum drive conditions.

When an electric vehicle is travelling in a straight line with maximum driving force under full load, the drive axle not only needs to bear the body’s own gravity and the weight of the motor and reducer, but also has to resist the shear stresses exerted by the driving force from the maximum torque output of the motor. The axle housing is analyzed under the maximum traction load condition, ignoring the effects of lateral forces and other complex road conditions that may be encountered by the vehicle.

In this case, the vertical load on the leaf spring seat is:


$$F_{T}=\frac{k_{g}\times G}{2}$$ (3–5)

Where G is the gravity of the vehicle when it is fully loaded and, kg is the load transfer coefficient of the axle when the vehicle is in linear form takes the value of 1.2.

Rotational counter-torque applied to a single side of the integrated drive axle housing:


$$T=\frac{T_{\max}\times i\times \eta_{t}}{2}$$ (6)

The drive axle is subject to motor and reducer forces:


$$G_{1}=m_{1}\times g$$ (7)

$T_{max}$ is the ultimate output torque of the drive axle motor, i is the gearbox ratio,$\eta_{t}$ is the transmission efficiency, and $G_{1}$ is the motor and gearbox gravity.

**Fig 7:**
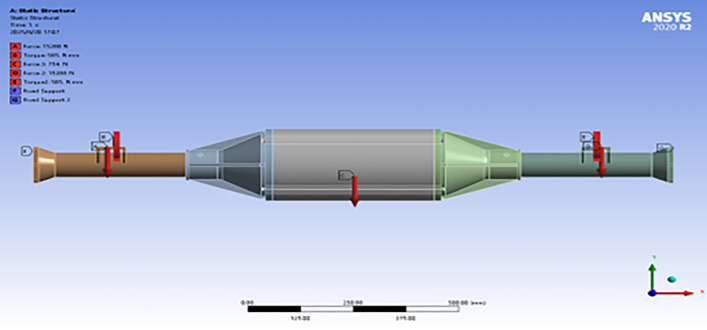
Constraints and loads applied under maximum drive conditions.

Setting load constraints: constrains the movement in the X, Y, Z, direction and rotation in the X, Y direction of the two ends of the integrated drive axle housing, so that displacement and turn to cannot occur at that end point. As shown in Fig. 7.

Setting the loading load: after bringing the parameters into the formulae for calculation, it is learnt that in this particular case, a force of 15288N is to be applied vertically downwards in the positive direction of the Y-axis of the spring steel plate seat, while a torque of 585N is to be applied at the spring seat, and a force of 754N is to be applied at the position of center of gravity of the axle housing. This is shown in Fig 7.

**Fig 8:**
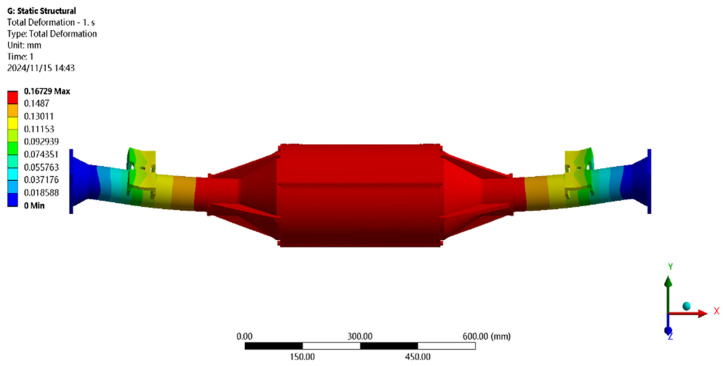
Displacement cloud of maximum driving force working condition.

**Fig 9:**
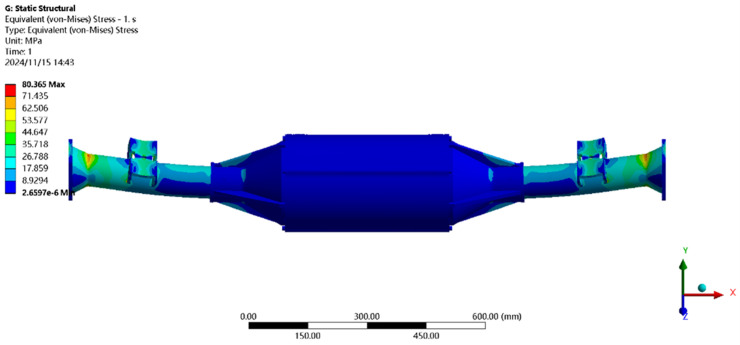
Stress cloud of maximum driving force working condition.

Using finite element to solve for the maximum driving force condition, the results of the integrated drive axle shell finite element simulation analysis stress and displacement cloud are obtained as shown in Figs 8 and 9.

In the analysis results of the maximum driving force working condition displacement cloud diagram in Fig 8, it can be seen that the integrated drive axle shell displacement deformation gradually increases from both ends to the middle of the shell. The maximum deformation position in the bridge shell body motor and reducer position, the maximum deformation of about 0.167 mm, the variable per meter wheelbase is 0.167/1.52 ≈ 0.111 mm/m, much smaller than the national regulations of 1.5 mm/m, which is determined to meet the steel strength.

From Fig 9 maximum driving force working condition stress cloud analysis results can be seen integrated drive axle shell maximum stress is located in the half-axle casing at both ends of the constraints, where the maximum stress position of the peak von Mises stress of 80.37 MPa, less than the integrated drive axle shell material 40Cr yield strength of 785 MPa. in other areas of the stress value is lower, are in line with the requirements of the performance specifications of the electric vehicle drive axle, which determines that the drive axle meets the strength requirements. The drive axle is determined to meet the strength requirements.

#### Emergency stop conditions.

In the case of emergency braking under full load, the axle plate spring is subjected to the vertical force G and the self-gravitational force of the motor and reducer G1 in the fully loaded state of the vehicle, as well as the braking force M of the vehicle on the ground.

The vertical force on the steel plate spring seat:


$$F_{V}=\frac{k_{D}\times G}{2}$$ (3–8)

Where, k_D_ is the mass transfer coefficient of the car during braking takes the value of 0.7.The size of braking torque is:


$$T_{L}=\frac{k_{D}\times G\times \varphi \times r}{2}$$ (3–9)

Where, φ wheels on the ground between the coefficient of adhesion to take 0.7, r for the rolling radius of the wheel

**Fig 10:**
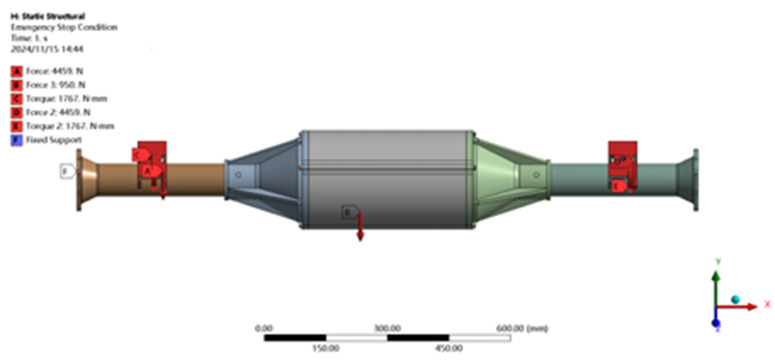
Constraint and load application in emergency stop condition.

Setting load constraints: constrains the movement in the X, Y, Z, direction and rotation in the X, Y direction of the two ends of the integrated drive axle housing, so that displacement and turn to cannot occur at that end point. As shown in Fig 10.

Setting the loading load: after bringing the parameters into the formulae for calculation, it is learnt that in this particular case, a force of 4459 N vertically downward is to be applied in the Y-axis positive direction at the spring steel plate seat, while a force of 950 N is to be applied at the position of center of gravity of the axle housing. It is also necessary to apply a braking torque of 1767N at the steel plate spring seat. This is shown in Fig 10.

**Fig 11:**
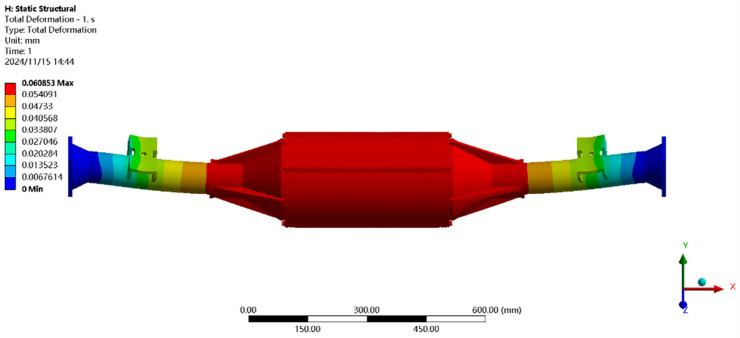
Displacement cloud of emergency stop condition.

**Fig 12:**
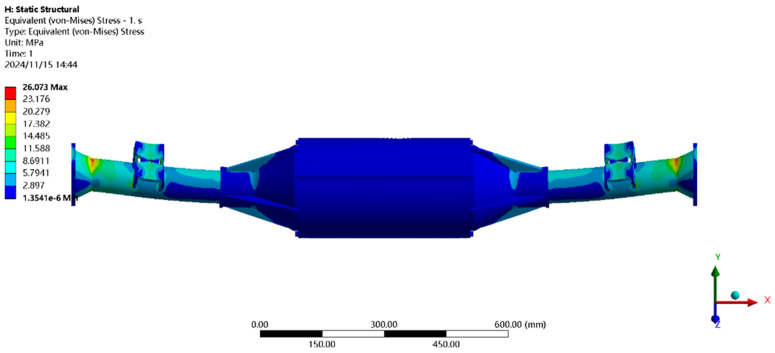
Emergency stop condition stress map.

Using finite element to solve for the emergency stop condition, the results of the integrated drive axle housing finite element simulation and analysis of the stress and displacement cloud are obtained as shown in Figs 11 and 12.

In the analysis results of the emergency stop condition displacement cloud diagram in Fig 11, it can be seen that the integrated drive axle shell displacement deformation gradually increases from both ends to the middle of the shell. The maximum deformation position in the bridge shell body motor and reducer position, the maximum deformation of about 0.06 mm, per meter wheelbase variable for 0.06/1.52 ≈ 0.04 mm/m, much smaller than the national regulations of 1.5 mm/m, which is determined to meet the steel strength.

From the results of the stress cloud analysis of the emergency stop condition in Fig 12, it can be seen that the maximum stress of the integrated drive axle shell is located in the half-axle casing at both ends of the constraints, in which the maximum stress position of the stress is 26.08MPa, less than the yield strength of the integrated drive axle shell material 40Cr 785MPa. in other areas of the stress value is lower, are in line with the requirements of the performance specifications of the EV drive axle, which is determined to be the drive axle meets the strength requirements. The drive axle is determined to meet the strength requirements.

#### Maximum lateral force conditions.

Pure electric vehicles are subjected to lateral forces generated when making sharp turns under changing road conditions. When the vehicle is subjected to the maximum lateral force limit condition, the axle not only needs to load the entire axle weight G of the vehicle and the weight G1 of the motor and gearbox, but also has to withstand the tangential force Fp.

Let,${\text{F}}_{\text{L}}$,${\text{F}}_{\text{R}}$ be the reaction forces given by the ground to the left and right wheels, respectively, when the car slips to the right: when ${\text{F}}_{\text{L}}$=0, then ${\text{F}}_{\text{R}}$ Calculate the formula:


$$F_{R}=\frac{G\times g}{2}$$ (10)

The lateral reaction force given by the ground to the inner and outer drive wheels is ${\text{F}}_{\text{T}}$, calculated by the formula:


$$F_{T}=F_{R}\times \varphi_{1}$$ (11)

Where, φ the coefficient of adhesion between the wheel and the ground is taken as 0.7.

The torque of the drive axle housing is generated by the tangential force as ${\text{T}}_{\text{R}}$, calculated by the formula:


$$T_{R}=F_{T}\times r$$ (12)

Where, r is the rolling radius of the wheel.

**Fig 13:**
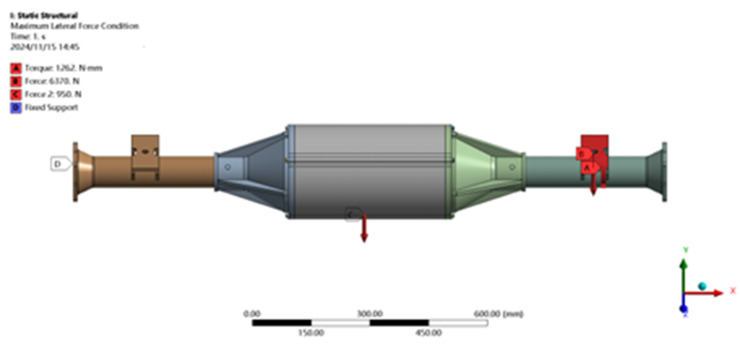
Constraint and load application for maximum lateral force condition.

Setting load constraints: constrains the movement of the integrated drive axle housing ends in the X, Y, and Z, directions and the rotation in the X and Y directions, so that no displacement or turn to can occur at that end point. As shown in Fig 13.

Setting loading load: After bringing the parameters into the formula calculation, it is known that in this particular working condition, a vertically downward force of 6370N is to be applied in the positive direction of the Y-axis of the right-hand side steel plate spring seat, while a moment of 1262N is to be applied at the right-hand side steel plate spring seat, and a force of 950N is to be applied at the centre of gravity position of the axle housing. As shown in Fig 13.

**Fig 14:**
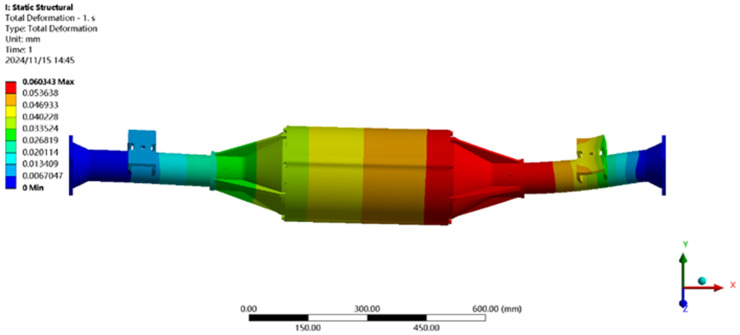
Maximum lateral force displacement map.

**Fig 15:**
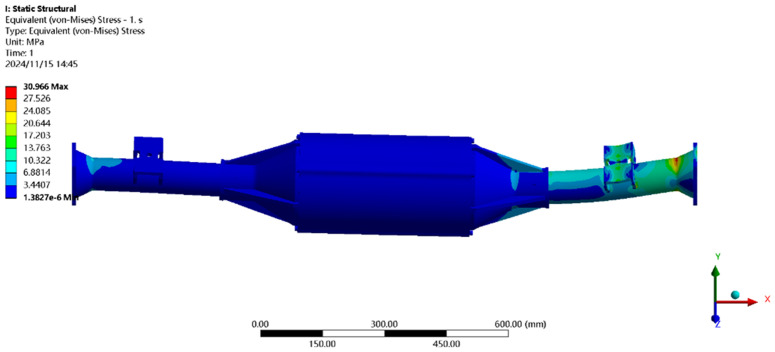
Stress cloud of maximum transverse force.

The maximum transverse force condition is solved using finite elements, and the results of the integrated drive axle shell finite element simulation analysis stress and displacement clouds are obtained as shown in Figs 14 and 15.

In the analysis results of the maximum transverse force working condition displacement cloud diagram in Fig 14, it can be seen that the integrated drive axle housing displacement deformation is mainly in the right side of the steel plate spring seat and the right side of the end cover position. The maximum deformation location from the integrated drive axle housing right end cover, the maximum deformation of about 0.06 mm, per meter wheelbase variable for 0.06/1.52 ≈ 0.04 mm/ m, far less than the national regulations of 1.5 mm/ m, which is determined to meet the steel strength.

From Fig 15 maximum transverse force working condition stress cloud analysis results can be seen integrated drive axle shell maximum stress is located in the right side of the half-axle casing steel plate spring seat near the location of the maximum stress of 30.99 MPa, less than the integrated drive axle shell material 40Cr yield strength of 785 MPa. in other areas of the stress value is lower, are in line with the requirements of the performance specification of the EV drive axle, thus determining that the drive axle meets the strength requirements. Thus, it is determined that the drive axle meets the strength requirements.

## Fatigue analysis of integrated drive axle housing

Based on the stress distribution results under four extreme working conditions, the Miner cumulative damage theory was adopted to predict the fatigue life of the integrated drive axle housing. Taking the key stress – bearing parts (such as the restraint of the half – shaft sleeve, near the leaf spring seat) as the analysis objects, the stress – time history under each working condition was extracted. Combining with the S – N curve of 40Cr steel after quenching and tempering treatment, it was calculated that the safety factor of the key parts under the maximum vertical load condition at 10⁶ cycles was 4.2. Referring to the cyclic load spectrum construction method proposed, the road excitation was transformed into variable – amplitude load. The number of stress cycles was counted by the rain – flow counting method. The results showed that the cumulative damage factor of Optimization Scheme 1 (body thickness 5 mm, end – cover 8 mm) within 10⁷ cycles was 0.18, and that of Scheme 2 (replacing the low – stress area with QT450) was 0.21, both of which were far lower than the failure threshold of 1.0. At the same time, for the problem of a slight increase in local stress caused by the reduction of wall thickness in Scheme 1, by increasing the fillet radius of the stress – concentration area from 5 mm to 8 mm, the fatigue safety factor of this part was maintained above 3.8 to ensure long – term use reliability. Comparing the fatigue performance of the two schemes, while Scheme 1 achieved a 12% weight reduction, the fatigue life of its key parts (2.3 × 10⁶ times) and that of Scheme 2 (1.9 × 10⁶ times) both met the design life requirement of 1.5 × 10⁶ times for commercial vehicle drive axles, verifying the long – term performance stability of the lightweight – optimized structure under cyclic loads.

### Finite element modal analysis of integrated drive axle housing

To ensure safe vehicle operation, if the road excitation frequency is close to or coincides with the intrinsic frequency of the drive axle housing, structural resonance will be triggered. Resonance may lead to deformation or damage of the vehicle structure, increasing the risk of traffic accidents. Therefore, the use of modal analysis is necessary to identify the intrinsic frequency and vibration pattern of the drive axle, and thus effectively avoid the safety problems caused by resonance.

### Introduction to the principles of modal analysis

Modal analysis is to consider the complex vibration of a structure as a linear combination of all orders of single-degree-of-freedom vibration. Each order of intrinsic mode corresponds to an intrinsic frequency, which describes the relative amplitude and phase relationship between points when the structure vibrates at that frequency. Low-order modes correspond to low-frequency vibrations, while high-order modes correspond to high-frequency vibrations.

### Modal analysis of integrated drive axle housing

**Fig 16:**
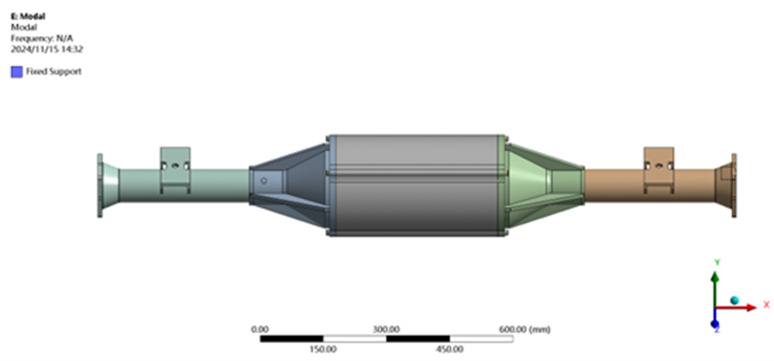
Modal boundary constraints.

Before performing the Finite Element Method (FEM) modal analysis, the axle is fixed constrained according to the reality as shown in Fig 16.

**Fig 17:**
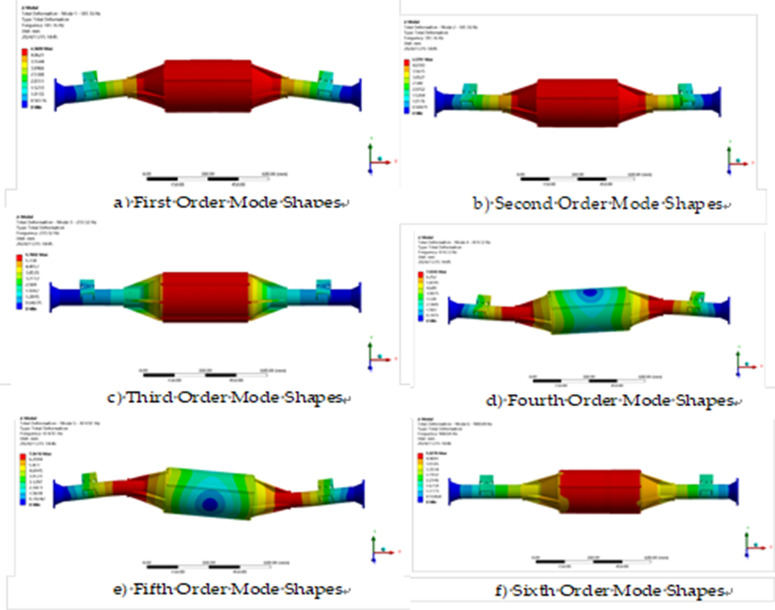
Vibration patterns of the first six sets of modal analysis.

The constrained model is solved by modal analysis using finite element analysis software and the first six orders of intrinsic frequency and vibration pattern maps are obtained as shown in Table 3 and Fig 17.

**Table 3:** First six orders of intrinsic frequency.

Number of steps	Natural frequency/Hz
First order	181.1
Second order	180.8
Third order	233.13
Fourth order	473.51
Fifth order	473.72
Sixth order	906.82

The modal analysis results show that the intrinsic vibration frequency of the integrated drive axle shell is between 180.8 Hz and 906.82 Hz. In the lower frequency band, the bridge shell mainly exhibits vibration in the vertical direction; while at higher frequencies, not only vertical vibration, but also torsional action is accompanied.

The vibration frequency caused by the road surface is generally between 0 and 50 Hz [35], while the vibration frequency of the direct-drive bridge in the first order mode is 180.8 Hz, and the other higher-order intrinsic frequencies are greater than the first order. Therefore, the axle will not resonate during actual operation.

### Lightweight symmetrical design of integrated drive axle housing

With the rapid development of electric vehicle technology, increasing range and energy efficiency are increasingly becoming the goals pursued by the industry. Especially in the context of environmental protection and energy shortage, the lightweight design of the drive axle, as an important part of the vehicle, is particularly critical, as it can reduce the weight of the vehicle in order to extend the battery range and improve the efficiency of energy use. Since the drive axle is directly related to the vehicle’s carrying capacity. Therefore, in the lightweight design of the drive axle at the same time to ensure sufficient strength. At present, the lightweight design of the drive axle mainly has the choice of high-strength low-density materials and structural size optimization as the main lightweight design methods. This paper also focuses on these two lightweight design methods, the specific implementation process is as follows:

(1) Selected dimensional structural modifications to the model or replacement of appropriate parts with lighter materials.
(2) Conduct finite element static analysis of the modified model under typical working conditions to confirm whether the stiffness and strength requirements are met.
(3) Conduct finite element modal analysis on the modified model to confirm whether the intrinsic frequency of the frame and the external stimulus frequency will overlap and resonance phenomenon occurs.
(4) Based on the results of the analysis, a comprehensive comparison of the various options is made to determine the final optimization plan.

#### Optimization scenario one.

**Fig 18:**
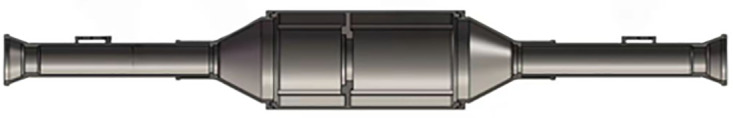
Model optimization structure selection diagram.

According to the results of the previous analysis, the overall maximum displacement and stress values of the drive axle housing are much smaller than the permissible values, so lightweighting can be achieved by reducing the overall structural thickness dimensions. In this scheme, the thickness dimensions of the half shaft, end cover, body and spring seat as shown in Fig. 18 will be appropriately thinned to achieve lightweighting, and the optimized dimensions are shown in Table 4.

**Table 4:** Dimensional optimization comparison chart.

Name	Thickness before optimization	Optimized thickness
Half Shaft	8mm	6mm
End cap	6mm	5mm
Subject	10mm	5mm
Spring seat	8mm	6mm

#### Analysis of working conditions.

The model after lightweight design is imported into finite element analysis software for finite element static analysis of four typical working conditions, with the same load application and boundary constraints as before optimization.

**Fig 19:**
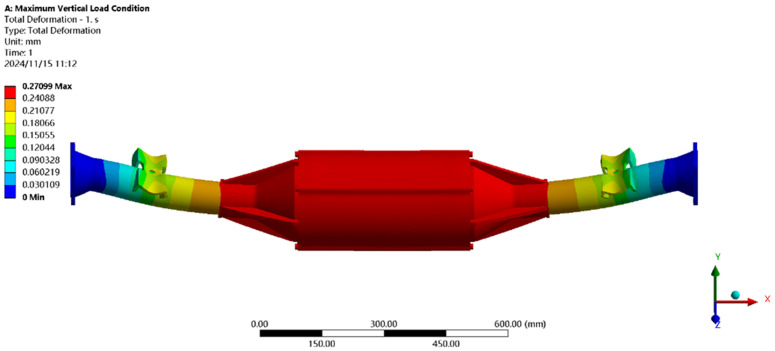
Displacement cloud of maximum vertical loads.

Fig 19 shows the displacement under maximum vertical load: the maximum deformation (≈0.27 mm) occurs at the motor and reducer position, with a per-meter wheelbase deformation of ≈0.177 mm/m, well below the national standard of 1.50 mm/m, meeting strength requirements.

**Fig 20:**
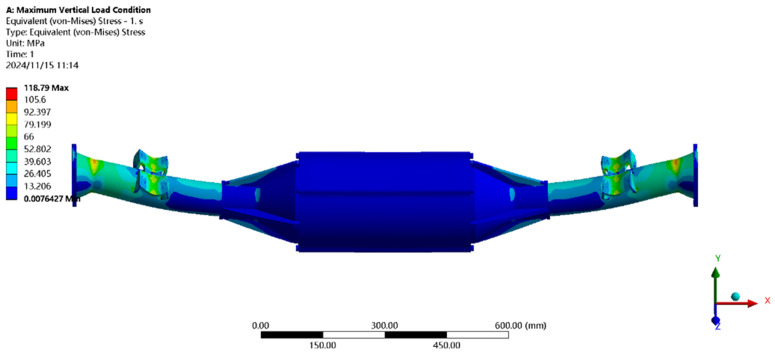
Maximum vertical load stress clouds.

Fig 20 shows the stress distribution under maximum vertical load: the peak stress (118.79MPa) is near the leaf spring seat of the half-shaft casing, far below the 40Cr yield strength (785MPa). All regions meet EV drive axle performance specifications.

**Fig 21:**
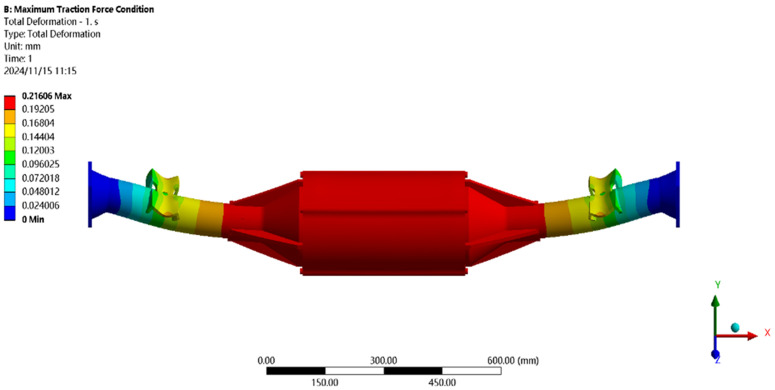
Displacement cloud of maximum driving force working condition.

Fig 21 shows the displacement under maximum driving force: the maximum deformation (≈0.21 mm) occurs at the motor and reducer position, with a per-meter wheelbase deformation of ≈0.14 mm/m, below the 1.50 mm/m standard.

**Fig 22:**
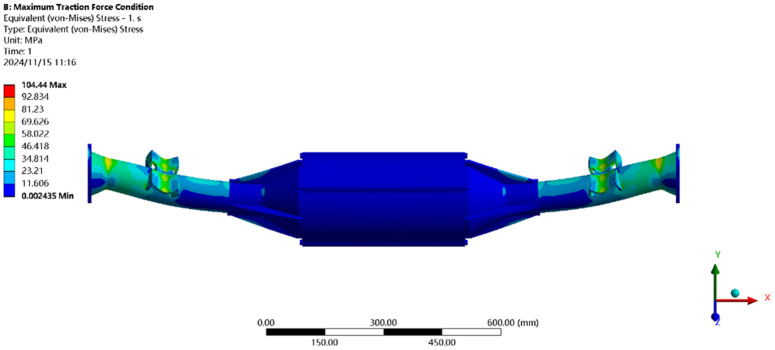
Maximum driving force working condition stress map.

Fig 22 shows the stress under maximum driving force: the peak stress (104.44MPa) is near the leaf spring seat, below the 40Cr yield strength (785MPa), meeting all performance requirements.

**Fig 23:**
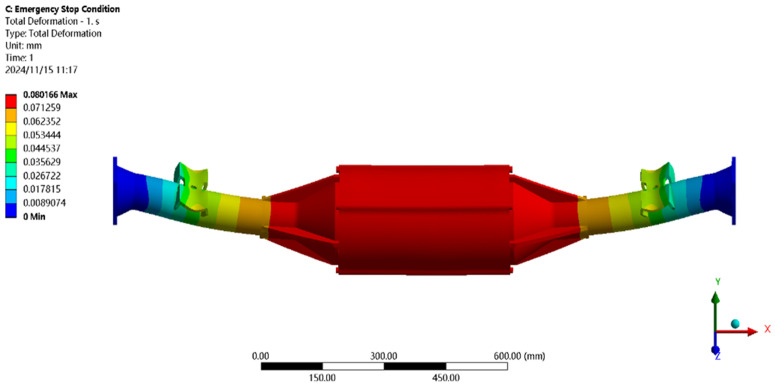
Displacement cloud of emergency stop condition.

In Fig 23 the results of the displacement cloud analysis under emergency stopping conditions can be seen in the integrated drive axle housing maximum deformation position in the axle housing body motor and reducer position, the maximum deformation of about 0.08 mm, per meter wheelbase variable for 0.08/1.52 ≈ 0.05 mm/m, far less than the national regulations of 1.50 mm/m, which was determined to meet the strength of the steel.

**Fig 24:**
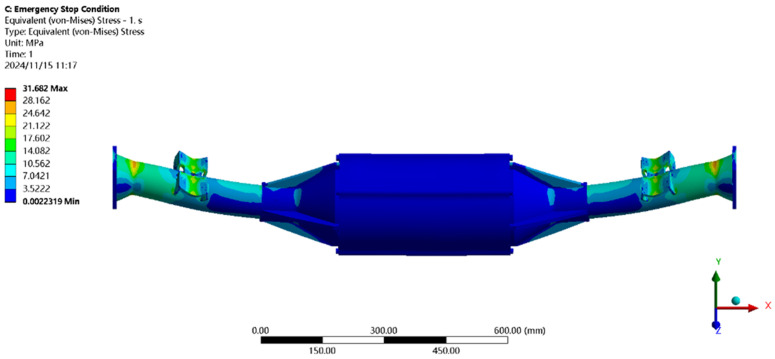
Emergency stop condition stress map.

In Fig 24, the results of stress cloud analysis under emergency stopping conditions show that the maximum stress of the integrated drive axle shell is located near the half-axle casing steel plate spring seat, in which the maximum stress position of the stress is 31.68MPa, which is smaller than the yield strength of the integrated drive axle shell material 40Cr of 785MPa. Other areas of stress values are lower, are in line with the requirements of the performance specifications of the electric vehicle drive axle, and therefore It is determined that the drive axle meets the strength requirements.

**Fig 25:**
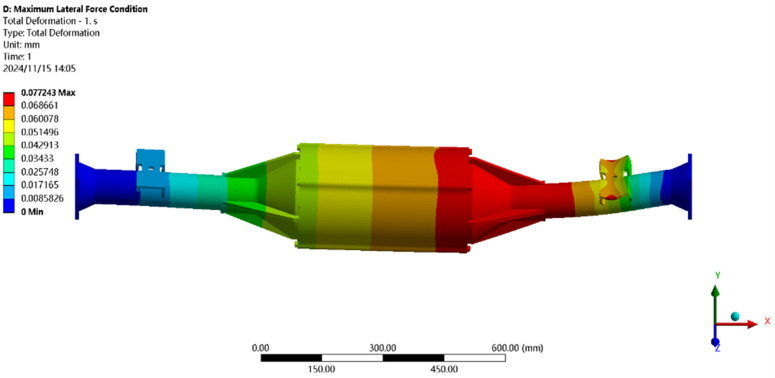
Displacement cloud for maximum lateral force condition.

In Fig 25 the maximum transverse force working conditions of the displacement map analysis results can be seen in the integrated drive axle shell maximum deformation location in the axle shell body motor and reducer position, the maximum deformation of about 0.077 mm, per meter wheelbase of the variable is 0.077/1.52 ≈ 0.05 mm/m, much smaller than the national regulations of 1.50 mm/m, which was determined to meet the strength of the steel.

**Fig 26:**
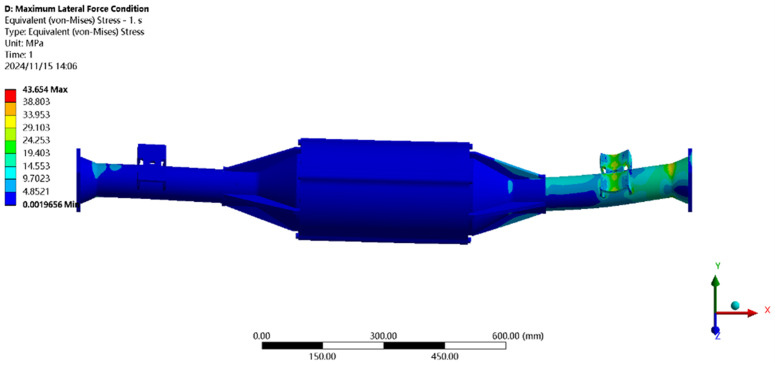
Maximum transverse force working condition stress clouds.

In Fig 26, the maximum transverse working conditions of the stress cloud analysis results can be seen in the integrated drive axle shell maximum stress is located in the half shaft casing steel plate spring seat near the location of the maximum stress of 43.65MPa, less than the integrated drive axle shell material 40Cr yield strength of 785MPa. other areas of stress values are lower, are in line with the requirements of the performance specifications of the electric vehicle drive axle, and therefore It is determined that the drive axle meets the strength requirements.

### Modal analysis

**Fig 27:**
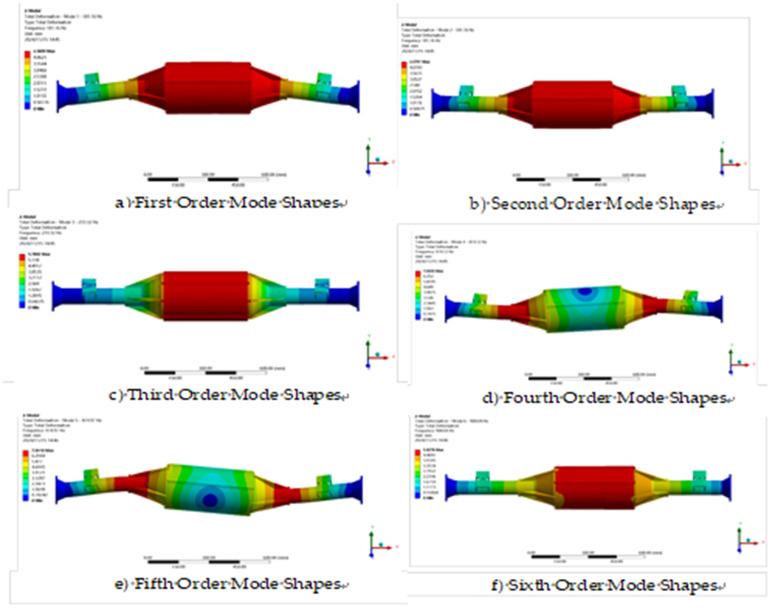
Shape of the first six orders of modal analysis.

A modal analysis was performed on the optimized model to determine the natural frequencies and vibration modes of the drive shaft, thereby avoiding safety issues caused by resonance. The optimization process utilized Latin hypercube sampling to generate 50 sets of sample points. A response surface model was employed to fit the relationship between the variables and the objective function, ultimately iterating to obtain the optimal solution: When the thickness of the main body is reduced by 50% (from 10 mm to 5 mm), the stress increase is controlled to 27.2% (from 93.36 MPa to 118.79 MPa), which remains far below the constraint threshold; The thickness reduction ratios for the end cap and spring seat are 16.7% and 25%, respectively, with stress increases both below 15%, while also validating feasibility through formal dimensional optimization algorithms. After optimization, the mass was reduced by 7.5 kg (12% weight reduction), and the safety factors under all operating conditions were ≥6.6 (785 MPa/118.79 MPa), meeting the redundancy design requirements for commercial vehicle drive axles. In the optimized model, modal analysis was performed using the same constraint conditions as before optimization. The first six vibration modes and frequencies obtained from the solution are shown in Table 5 and Fig 27.

**Table 5:** First six orders of intrinsic frequency.

Number of steps	Natural frequency/Hz
First order	192.72
Second order	192.72
Third order	258.95
Fourth order	486.20
Fifth order	486.50
Sixth Step	969.29

According to Table 5, the first six orders of inherent frequency table shows that the natural vibration frequency of the direct-connected drive bridge is between 192.27 Hz and 969.29 Hz. The vibration frequency caused by the road surface is generally between 0 and 50 Hz, while the vibration frequency of the direct-connected drive axle in the first-order mode is 192.7 Hz, and the other orders of intrinsic frequency are greater than the first order. Therefore, the axle will not resonate during actual operation.

### Quality assessment

The pre-optimized and post-optimized models were subjected to gravimetric assessment through the mass assessment tool in 3D modeling software respectively and the result obtained from the pre-optimized model was 62.56 kg and the result obtained from the post-optimized model was 55.06 kg. The weight of the optimized model has been reduced by 12% relative to the pre-optimized model with a reduction in the mass of 7.5 kg as shown in Table 6.

**Table 6:** Quality data before and after optimization.

	Before optimization	After Optimization	Decrease ratio
quantity	62.56 kg	55.06 kg	12%

### Optimization scenario two

After the static analysis prior to optimization, it can be observed that the stresses are mainly concentrated at the spring seats and half shafts, while the stress levels on both sides of the axle housing body and end caps are relatively low. In view of this, a lighter and slightly lower strength material is considered to replace the original axle housing body and end caps, and QT450 is chosen as the replacement material in this study. At the same time, in order to ensure the reliability of the structure, the half shaft and spring seat part will continue to use high strength 40Cr steel. The material selection and parameter setting of each component are shown in Table 7.

**Table 7:** Integrated drive axle housing material configuration.

Components	Materials	Modulus of elasticity/MPa	Poisson’s ratio	Yielding strength/MPa	Densities kg/m^3
Half Shaft	40Cr	208000	0.3	785	7845
End caps	QT450	169000	0.28	310	7000
Body	QT450	169000	0.28	310	7000

### Analysis of working conditions

The material-improved model is set up in finite element analysis software with the same load conditions and boundary constraints as before optimization, and an exhaustive finite element static analysis is carried out for four typical working conditions to ensure the reliability and effectiveness of the integrated drive axle housing.

**Fig 28:**
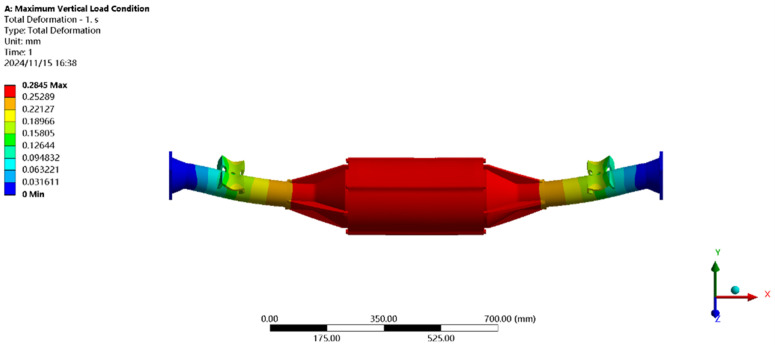
Displacement cloud for maximum vertical load condition.

In Fig 28 the maximum vertical working conditions of the displacement map analysis results can be seen in the integrated drive axle housing maximum deformation location in the axle housing body motor and reducer position, the maximum deformation of about 0.21 mm, per meter wheelbase variable for 0.21/1.52 ≈ 0.13 mm/m, far less than the national regulations of 1.50 mm/m, which was determined to meet the steel strength.

**Fig 29:**
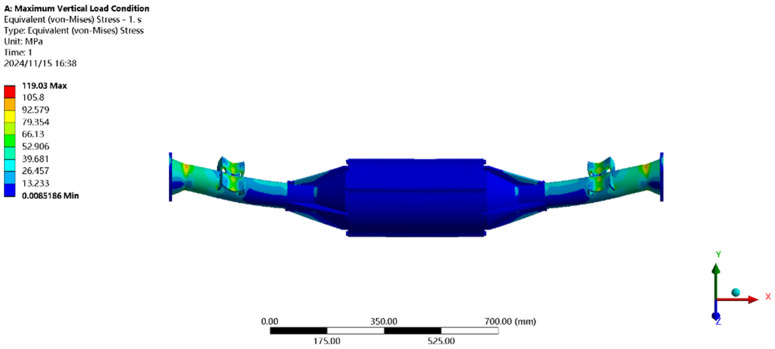
Maximum vertical loading condition stress map.

In Fig 29, the maximum vertical working conditions of the stress cloud analysis results can be seen in the integrated drive axle shell maximum stress is located in the half shaft casing steel plate spring seat near the location of the maximum stress of 92.85MPa, less than the integrated drive axle shell material 40Cr yield strength of 785MPa. other areas of stress values are lower, are in line with the requirements of the performance specifications of the electric vehicle drive axle, and therefore It is determined that the drive axle meets the strength requirements.

**Fig 30:**
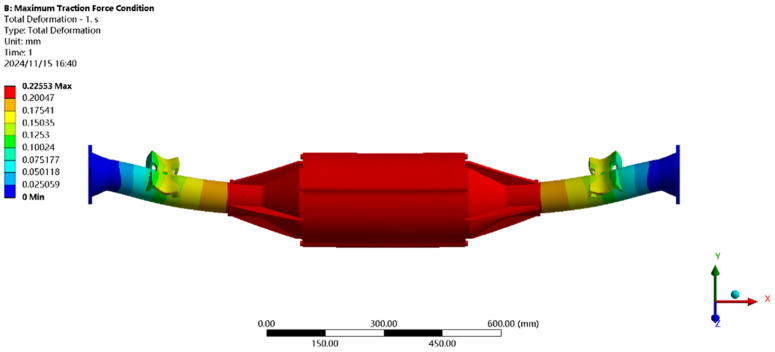
Displacement cloud of maximum driving force working condition.

In Fig 30 the maximum driving force working conditions of the displacement map analysis results can be seen in the integrated drive axle housing maximum deformation location in the axle housing body motor and reducer position, the maximum deformation of about 0.17 mm, per meter wheelbase variable for 0.17/1.52 ≈ 0.11 mm/m, far less than the national regulations of 1.50 mm/m, which was determined to meet the steel strength.

**Fig 31:**
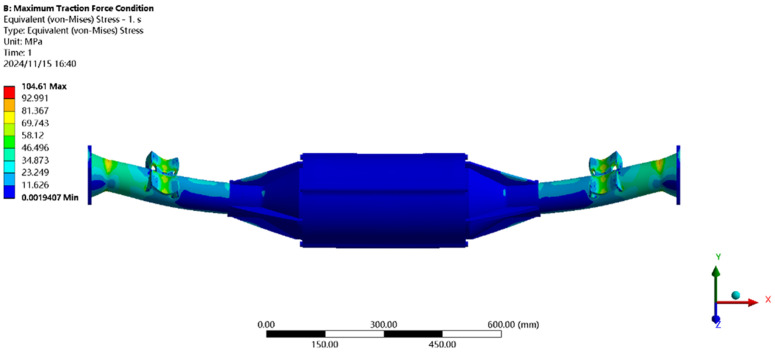
Stress cloud for maximum driving force condition.

In Fig 31 the maximum driving force working conditions of the stress cloud analysis results can be seen in the integrated drive axle shell maximum stress is located in the half-axle casing steel plate spring seat near the location of the maximum stress of 80.74MPa, far less than the integrated drive axle shell material 40Cr yield strength of 785MPa. other areas of stress values are lower, are in line with the requirements of the performance specifications of the EV drive axle, thus determining that the drive axle meets the strength requirements. It is thus determined that the drive axle meets the strength requirements.

**Fig 32:**
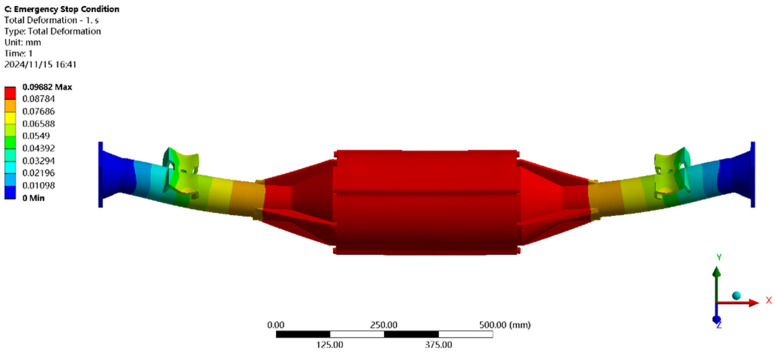
Emergency stop condition displacement map.

In Fig 32, the results of the displacement cloud analysis under emergency stopping conditions can be seen in the integrated drive axle housing maximum deformation location in the axle housing body motor and reducer position, the maximum deformation of about 0.06 mm, per meter wheelbase variable for 0.06/1.52 ≈ 0.04 mm/m, far less than the national regulations of 1.50 mm/m, which was determined to meet the strength of the steel.

**Fig 33:**
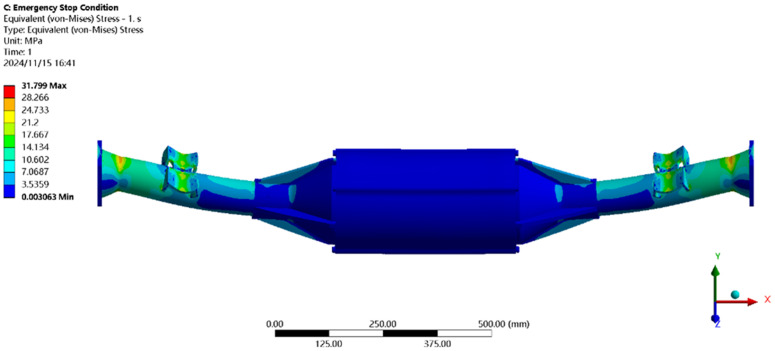
Emergency stop condition stress map.

In Fig 33 emergency stop condition stress map analysis results can be seen in the integrated drive axle shell maximum stress is located in the half-axle casing steel plate spring seat near the location of the maximum stress of 25.98MPa, far less than the integrated drive axle shell material 40Cr yield strength of 785MPa. other areas of stress values are lower, are in line with the requirements of the performance specifications of the EV drive axle, thus determining that the drive axle meets the strength requirements. It is thus determined that the drive axle meets the strength requirements.

**Fig 34:**
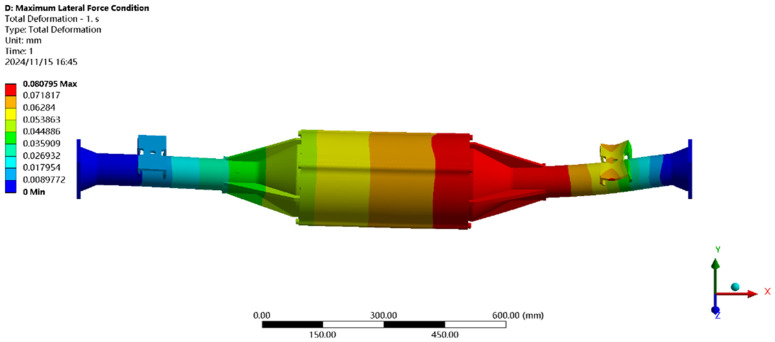
Displacement cloud at maximum lateral force condition.

In Fig 34 the maximum lateral force working conditions of the displacement cloud analysis results can be seen in the integrated drive axle shell maximum deformation location in the axle shell body motor and reducer position, the maximum deformation of about 0.07 mm, the average per meter wheelbase variable for 0.07/1.52 ≈ 0.05 mm/ m, much smaller than the provisions of our country’s 1.50 mm/m, which was determined to meet the strength of the steel.

**Fig 35:**
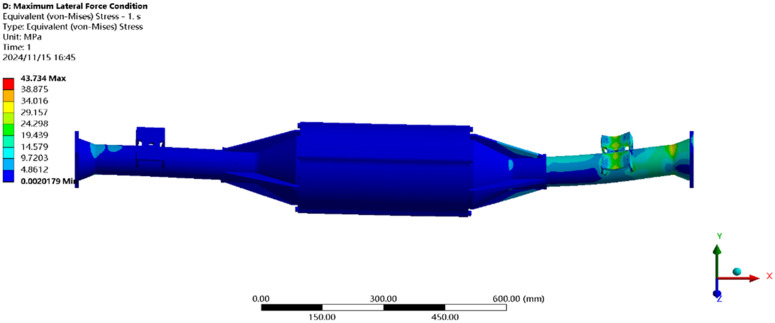
Stress map for maximum transverse force condition.

The maximum lateral load stress distribution diagram in Fig 35 shows that the integrated electric drive axle housing is subjected to the highest stress in the area of the leaf spring seat of the half shaft casing, with a peak stress of 43.65 MPa. This value is far below the yield limit of the 40Cr material used to manufacture drive axle housings, 785 MPa. The other parts of the axle housing have relatively small stress values, and are in line with the performance specification for electric vehicle drive axles.. Therefore, it can be concluded that the design strength of the drive axle meets the requirements.

### Modal analysis

**Fig 36:**
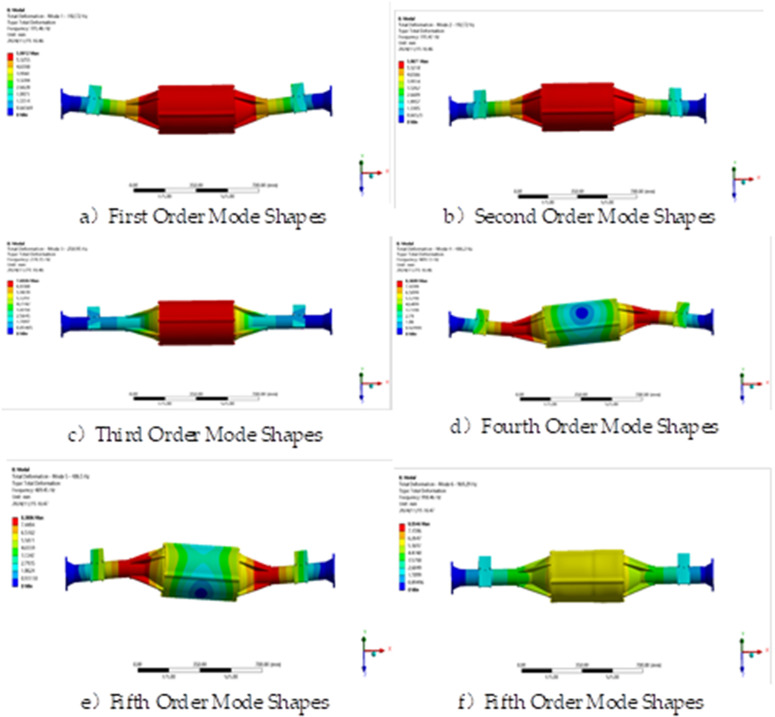
First six orders of modal shapes.

The modal analysis of the optimized model is carried out using the same constraint settings as before the optimization, and the first six orders of vibration patterns and frequencies obtained from the solution are shown in Table 8 and Fig 36.

**Table 8:** First six orders of intrinsic frequency for Option 2.

Number of steps	Neural frequency/Hz
First order	195.46
Second order	195.47
Third order	270.35
Fourth order	489.13
Fifth order	489.45
Sixth Step	998.46

According to the first six modes of Fig 36, the intrinsic vibration frequency of the inline drive axle ranges from 195.46 Hz to 998.46 Hz. Normally, the frequency range of vibration caused by road surface unevenness is from 0 to 50 Hz. In comparison, the first order mode of the inline electric drive axle presents an intrinsic frequency of 194.46 Hz, while the other order intrinsic frequencies are greater than the first order. Therefore, the axle does not resonate during actual operation.

### Quality assessment

The pre-optimized and post-optimized models were subjected to gravimetric assessment through the mass assessment tool in 3D modeling software, respectively, and the pre-optimized model yielded a result of 62.56 kg and the post-optimized model yielded a result of 57.48 kg. The weight of the optimized model has been reduced by 8.1% with respect to the pre-optimized model, resulting in a reduction of 5.08 kg in mass, as shown in Table 9.

**Table 9:** Quality data before and after optimization.

	Before optimization	After Optimization	Decrease ratio
Quantity	62.56 kg	57.48 kg	8.1%

### Comparison of optimization options

It can be seen from the results of static and modal analyses of the two optimized solutions that the structural strength of the two optimized solutions does not exceed the permissible stress level of the material under a variety of working conditions, and maintains a sufficient margin of safety. Meanwhile, the structural stiffness also meets the design requirements. The best optimized solution is determined by comparing the stress, deformation and weight reduction of the integrated drive axle housing before and after optimization.

### Data comparison of optimization results

The optimized finite element static analysis results of the two schemes are more than that, the optimized stresses of both schemes meet the strength requirement requirements, as shown in Table 10.

**Table 10:** Comparison of the displacement results of the two schemes for each working condition.

	Maximum vertical working conditions	Maximum driving force	Emergency stop condition	Maximum lateral force
Option I	0.27mm	0.21mm	0.08mm	0.12mm
Option 2	0.21mm	0.17mm	0.06mm	0.07mm
Permissible value	Displacement value/wheelbase > 1.5 mm			

The displacements of the stress analysis results of both optimized schemes satisfy the steel requirements, as shown in Table 11.

**Table 11:** Comparison of stress results for the two scenarios.

	Maximum vertical working conditions	Maximum driving force	Emergency stop condition	Maximum lateral force
Option I	118.79MPa	104.44MPa	31.68MPa	258.57MPa
Option 2	92.854MPa	80.74MPa	25.98MPa	191.10MPa
Permissible value	< 785MPa			

The optimized finite element modal analysis results of the two schemes than, it can be seen that the intrinsic frequency of the two schemes are in the safe range, as shown in Table 12, the first order modal intrinsic frequency distribution of the two schemes is 192.72 Hz and 174.64 Hz, are not in the range of 0 ~ 50 Hz, the other order is also greater than the first order therefore the two schemes will not resonate.

**Table 12:** Comparison of inherent frequency results for the two schemes.

	First order/Hz	Second order/Hz	Third order/Hz	Fourth order/Hz	Fifth order/Hz	Sixth order/Hz
Option I	192.72	192.72	258.95	486.20	486.50	969.29
Option 2	195.46	195.47	270.35	489.13	489.45	998.46

A comparison of the model weight assessment results for the two optimization scenarios is shown in Table 13.

**Table 13:** Quality assessment results comparing the two design options.

	Before optimization	After Optimization	Decrease ratio
Option I	62.56 kg	55.10 kg	12%
Option 2	62.56 kg	57.48 kg	8.1%

### Determination of the optimal optimization scheme

By comparing the above two optimization schemes, it can be found that both optimization schemes fully satisfy the requirements of structural strength and stiffness. Through modal analysis, it is further confirmed that the potential risk of resonance phenomenon is avoided in all orders of intrinsic frequency for both solutions. In terms of mass assessment, solution 1 shows a significant advantage with a 12% mass reduction compared to the pre-optimized design. Comparatively, the mass of option 2 is reduced by only 8.1%. Given that both solutions meet the structural stiffness and strength requirements and have a safe intrinsic frequency range, Option 1 was determined to be the more desirable lightweight solution after considering the mass reduction advantages.

## Conclusions

In this study, static finite element analysis and modal finite element analysis of the integrated drive axle housing are carried out for typical working conditions. This paper elaborates the application of two lightweight design solutions and identifies a better solution strategy through comparative analysis. The following are the conclusions of the work in the research engineering of the integrated drive axle housing studied in this paper:

(1) By reviewing and analyzing the cutting-edge literature at home and abroad, the latest scientific research dynamics and development trends on electric vehicle drive axles are obtained. It gives a more comprehensive understanding of the technological progress and future prospects in this field. Accordingly, this paper defines the specific direction of research and plans a detailed technology roadmap.
(2) With reference to the existing models on the market, a simplified integrated drive axle housing model is established and material information is determined by 3D modeling software according to the main parameters of the model.
(3) In the lightweight design of the drive axle, sufficient strength is ensured. Through finite element analysis software, the 40Cr steel integrated drive axle housing model was meshed, and a mesh sensitivity analysis was performed to verify convergence. The analysis confirmed that 5 mm (high-stress areas) and 8.5 mm (low-stress areas) mesh sizes ensure result reliability (variation <0.5% with finer meshes).
(4) For the integrated drive axle housing, static mechanical evaluations under four main operating conditions were executed in this study. The data obtained show that the stiffness and strength of the structure meets the preset performance criteria in all considered scenarios. Through further constrained modal analysis, the first six natural vibration frequencies of the structure were successfully identified and accordingly analyzed to confirm that the drive axle would not experience resonance problems under normal operating conditions, ensuring its operational stability.
(5) Two optimization schemes were selected, namely, modifying the thickness dimension of the drive axle housing and replacing the material of the less stressed parts with a material of lower density and strength, QT450, and the same four operating conditions and modal analysis as before optimization were carried out again.
(6) After a detailed comparison of the results of the two optimization schemes, it is concluded that under the condition of meeting the safety standards, Scheme 1 is significantly better than Scheme 2 in terms of lightweighting effect, so we finally determine Scheme 1 as the best optimization scheme.

This study focuses on the lightweight design and static modal analysis of integrated drive axle shells, but the lack of a large number of experimental simulation verification has led to a certain limit to the scope and depth of this study, future research work will be aimed at the development of finite element analysis software to improve the accuracy of the model analysis, and try to carry out more field tests and simulation verification to ensure the consistency of the simulation analysis and the actual performance.

## Supporting information

S1 Data (DOCX)
