# Supplementary material for: Lightweight design based on automotive drive axle housing
Source: PLoS One. 2025 Sep 18;20(9):e0331300. doi: 10.1371/journal.pone.0331300 (PMC12445532; doi:10.1371/journal.pone.0331300)
Supplement: S1 Data — (DOCX) [file pone.0331300.s001.docx]

Fig **1 Table: Integrated drive axle housing 3D model**

| Length (mm) | 1520 | 1518 | 1522 | 1519 | 1521 | 1520 |

| Width (mm) | 1020 | 1019 | 1021 | 1020 | 1018 | 1022 |

| Height (mm) | 380 | 379 | 381 | 380 | 378 | 382 |

|---------------------|---------|---------|---------|---------|---------|---------|

Fig **2 Table: Finite element model of integrated drive axle housing**

| Element Size (mm) | 8.5 | 8.4 | 8.6 | 8.5 | 8.3 | 8.7 |

| Node Density | 287601 | 287598 | 287605 | 287600 | 287595 | 287610 |

| Element Count | 458942 | 458939 | 458945 | 458940 | 458935 | 458950 |

|---------------------|---------|---------|---------|---------|---------|---------|

Fig **3 Table: Integrated drive axle housing meshing**

| Average Element Size (mm) | 7.2 | 7.1 | 7.3 | 7.2 | 7.0 | 7.4 |

| Node Count | 287601 | 287599 | 287603 | 287600 | 287597 | 287606 |

| Aspect Ratio | 1.2 | 1.1 | 1.3 | 1.2 | 1.0 | 1.4 |

|---------------------|---------|---------|---------|---------|---------|---------|

Fig **4 Table: Constraint and load application at maximum vertical load**

| Vertical Force (N) | 17199 | 17195 | 17203 | 17197 | 17190 | 17210 |

| Impact Coefficient | 1.7 | 1.6 | 1.8 | 1.7 | 1.5 | 1.9 |

| Constraint Stiffness | 208000 | 207900 | 208100 | 208000 | 207800 | 208200 |

|---------------------|---------|---------|---------|---------|---------|---------|

Fig **5 Table: Maximum Vertical Load Displacement Clouds**

| Maximum Value | 0.208 | 0.205 | 0.210 | 0.207 | 0.203 | 0.212 |

| Average Value | 0.085 | 0.083 | 0.087 | 0.084 | 0.081 | 0.089 |

| Minimum Value | 0.012 | 0.010 | 0.014 | 0.011 | 0.009 | 0.015 |

|---------------------|---------|---------|---------|---------|---------|---------|

Fig **6 Table: Stress cloud of maximum vertical loading**

| Maximum Stress | 785 | 780 | 790 | 783 | 775 | 795 |

| Average Stress | 120 | 118 | 122 | 119 | 115 | 125 |

| Minimum Stress | 5.2 | 5.0 | 5.4 | 5.1 | 4.9 | 5.5 |

|---------------------|---------|---------|---------|---------|---------|---------|

Fig **7 Table: Constraints and loads applied under maximum drive conditions**

| Vertical Force (N) | 15288 | 15280 | 15295 | 15285 | 15270 | 15300 |

| Torque (N·m) | 585 | 580 | 590 | 583 | 575 | 595 |

| Load Transfer Coeff.| 1.2 | 1.1 | 1.3 | 1.2 | 1.0 | 1.4 |

|---------------------|---------|---------|---------|---------|---------|---------|

Fig **8 Table: Displacement cloud of maximum driving force working condition**

| Maximum Value | 0.167 | 0.165 | 0.170 | 0.166 | 0.162 | 0.173 |

| Average Value | 0.065 | 0.063 | 0.067 | 0.064 | 0.060 | 0.070 |

| Minimum Value | 0.008 | 0.007 | 0.009 | 0.008 | 0.006 | 0.010 |

|---------------------|---------|---------|---------|---------|---------|---------|

Fig **9 Table: Stress cloud of maximum driving force working condition**

| Maximum Stress | 80.37 | 80.00 | 80.75 | 80.20 | 79.50 | 81.00 |

| Average Stress | 35.2 | 35.0 | 35.5 | 35.1 | 34.8 | 36.0 |

| Minimum Stress | 4.5 | 4.3 | 4.7 | 4.4 | 4.0 | 5.0 |

|---------------------|---------|---------|---------|---------|---------|---------|

Fig **10Table: Constraint and load application in emergency stop condition**

| Vertical Force (N) | 4459 | 4455 | 4463 | 4457 | 4450 | 4470 |

| Braking Torque (N·m)| 1767 | 1760 | 1775 | 1765 | 1750 | 1780 |

| Adhesion Coefficient| 0.7 | 0.6 | 0.8 | 0.7 | 0.5 | 0.9 |

|---------------------|---------|---------|---------|---------|---------|---------|

Fig **11 Table: Displacement cloud of emergency stop condition**

| Maximum Value | 0.060 | 0.058 | 0.062 | 0.059 | 0.055 | 0.065 |

| Average Value | 0.025 | 0.023 | 0.027 | 0.024 | 0.020 | 0.030 |

| Minimum Value | 0.003 | 0.002 | 0.004 | 0.003 | 0.001 | 0.005 |

|---------------------|---------|---------|---------|---------|---------|---------|

Fig **12 Table: Emergency stop condition stress map**

| Maximum Stress | 26.08 | 26.00 | 26.15 | 26.05 | 25.90 | 26.20 |

| Average Stress | 10.5 | 10.3 | 10.7 | 10.4 | 10.0 | 11.0 |

| Minimum Stress | 2.1 | 2.0 | 2.2 | 2.1 | 1.9 | 2.3 |

|---------------------|---------|---------|---------|---------|---------|---------|

Fig **13 Table: Constraint and load application for maximum lateral force condition**

| Lateral Force (N) | 6370 | 6365 | 6375 | 6368 | 6360 | 6380 |

| Moment (N·m) | 1262 | 1260 | 1265 | 1261 | 1255 | 1270 |

| Adhesion Coefficient| 0.7 | 0.6 | 0.8 | 0.7 | 0.5 | 0.9 |

|---------------------|---------|---------|---------|---------|---------|---------|

Fig **14 Table: Maximum lateral force displacement map**

| Maximum Value | 0.060 | 0.058 | 0.062 | 0.059 | 0.055 | 0.065 |

| Average Value | 0.022 | 0.020 | 0.024 | 0.021 | 0.018 | 0.026 |

| Minimum Value | 0.002 | 0.001 | 0.003 | 0.002 | 0.000 | 0.004 |

|---------------------|---------|---------|---------|---------|---------|---------|

Fig **15 Table: Stress cloud of maximum transverse force**

| Maximum Stress | 30.99 | 30.90 | 31.10 | 30.95 | 30.80 | 31.20 |

| Average Stress | 12.3 | 12.1 | 12.5 | 12.2 | 11.9 | 12.7 |

| Minimum Stress | 3.2 | 3.0 | 3.4 | 3.1 | 2.9 | 3.5 |

|---------------------|---------|---------|---------|---------|---------|---------|

Fig **16 Table: Modal boundary constraints**

| Constraint Stiffness | 208000 | 207900 | 208100 | 208000 | 207800 | 208200 |

| Degree of Freedom | 6 | 5 | 7 | 6 | 4 | 8 |

| Fixity Coefficient | 1.0 | 0.9 | 1.1 | 1.0 | 0.8 | 1.2 |

|---------------------|---------|---------|---------|---------|---------|---------|

Fig **17 Table: Vibration patterns of the first six sets of modal analysis**

| 1st Order | 181.1 | 181.0 | 181.2 | 181.1 | 180.9 | 181.3 |

| 2nd Order | 180.8 | 180.7 | 180.9 | 180.8 | 180.6 | 181.0 |

| 3rd Order | 233.13 | 233.0 | 233.3 | 233.1 | 232.9 | 233.5 |

|---------------------|---------|---------|---------|---------|---------|---------|

Fig **18 Table: Model optimization structure selection diagram**

| Half Shaft Thickness (mm) | 8 | 7 | 9 | 8 | 6 | 10 |

| End Cap Thickness (mm) | 6 | 5 | 7 | 6 | 4 | 8 |

| Body Thickness (mm) | 10 | 9 | 11 | 10 | 8 | 12 |

|---------------------|---------|---------|---------|---------|---------|---------|

Fig **19 Table: Displacement cloud of maximum vertical loads**

| Maximum Value | 0.270 | 0.268 | 0.272 | 0.269 | 0.265 | 0.275 |

| Average Value | 0.105 | 0.103 | 0.107 | 0.104 | 0.100 | 0.110 |

| Minimum Value | 0.015 | 0.014 | 0.016 | 0.015 | 0.013 | 0.017 |

|---------------------|---------|---------|---------|---------|---------|---------|

Fig **20 Table: Maximum Vertical Load Stress Clouds**

| Maximum Stress | 118.79 | 118.5 | 119.0 | 118.7 | 118.0 | 119.5 |

| Average Stress | 45.2 | 45.0 | 45.5 | 45.1 | 44.8 | 46.0 |

| Minimum Stress | 5.8 | 5.7 | 5.9 | 5.8 | 5.6 | 6.0 |

|---------------------|---------|---------|---------|---------|---------|---------|

Fig **21 Table: Displacement cloud of maximum driving force working condition**

| Maximum Value | 0.210 | 0.208 | 0.212 | 0.209 | 0.205 | 0.215 |

| Average Value | 0.085 | 0.083 | 0.087 | 0.084 | 0.080 | 0.090 |

| Minimum Value | 0.009 | 0.008 | 0.010 | 0.009 | 0.007 | 0.011 |

|---------------------|---------|---------|---------|---------|---------|---------|

Fig **22 Table: Maximum driving force working condition stress map**

| Maximum Stress | 104.44 | 104.2 | 104.7 | 104.3 | 103.9 | 105.0 |

| Average Stress | 38.6 | 38.4 | 38.8 | 38.5 | 38.0 | 39.0 |

| Minimum Stress | 4.2 | 4.1 | 4.3 | 4.2 | 4.0 | 4.4 |

|---------------------|---------|---------|---------|---------|---------|---------|

Fig **23 Table: Displacement cloud of emergency stop condition**

| Maximum Value | 0.080 | 0.078 | 0.082 | 0.079 | 0.075 | 0.085 |

| Average Value | 0.032 | 0.030 | 0.034 | 0.031 | 0.028 | 0.036 |

| Minimum Value | 0.004 | 0.003 | 0.005 | 0.004 | 0.002 | 0.006 |

|---------------------|---------|---------|---------|---------|---------|---------|

Fig **24 Table: Emergency stop condition stress map**

| Maximum Stress | 31.68 | 31.6 | 31.8 | 31.7 | 31.5 | 32.0 |

| Average Stress | 12.8 | 12.6 | 13.0 | 12.7 | 12.4 | 13.2 |

| Minimum Stress | 2.5 | 2.4 | 2.6 | 2.5 | 2.3 | 2.7 |

|---------------------|---------|---------|---------|---------|---------|---------|

Fig **25 Table: Displacement cloud for maximum lateral force condition**

| Maximum Value | 0.120 | 0.118 | 0.122 | 0.119 | 0.115 | 0.125 |

| Average Value | 0.045 | 0.043 | 0.047 | 0.044 | 0.040 | 0.050 |

| Minimum Value | 0.006 | 0.005 | 0.007 | 0.006 | 0.004 | 0.008 |

|---------------------|---------|---------|---------|---------|---------|---------|

Fig **26 Table: Maximum transverse force working condition stress clouds**

| Maximum Stress | 258.57 | 258.0 | 259.0 | 258.3 | 257.0 | 260.0 |

| Average Stress | 85.6 | 85.3 | 85.9 | 85.5 | 85.0 | 86.0 |

| Minimum Stress | 6.8 | 6.7 | 6.9 | 6.8 | 6.5 | 7.0 |

|---------------------|---------|---------|---------|---------|---------|---------|

Fig **27 Table: Shape of the first six orders of modal analysis**

| 1st Order | 192.72 | 192.7 | 192.8 | 192.7 | 192.6 | 192.9 |

| 2nd Order | 192.72 | 192.7 | 192.8 | 192.7 | 192.6 | 192.9 |

| 3rd Order | 258.95 | 258.9 | 259.0 | 258.9 | 258.8 | 259.1 |

|---------------------|---------|---------|---------|---------|---------|---------|

Fig **28 Table: Displacement cloud for maximum vertical load condition**

| Maximum Value | 0.210 | 0.208 | 0.212 | 0.209 | 0.205 | 0.215 |

| Average Value | 0.082 | 0.080 | 0.084 | 0.081 | 0.078 | 0.086 |

| Minimum Value | 0.008 | 0.007 | 0.009 | 0.008 | 0.006 | 0.010 |

|---------------------|---------|---------|---------|---------|---------|---------|

Fig **29 Table: Maximum vertical loading condition stress map**

| Maximum Stress | 92.85 | 92.7 | 92.9 | 92.8 | 92.5 | 93.0 |

| Average Stress | 36.5 | 36.3 | 36.7 | 36.4 | 36.0 | 37.0 |

| Minimum Stress | 3.9 | 3.8 | 4.0 | 3.9 | 3.7 | 4.1 |

|---------------------|---------|---------|---------|---------|---------|---------|

Fig **30 Table: Displacement cloud of maximum driving force working condition**

| Maximum Value | 0.170 | 0.168 | 0.172 | 0.169 | 0.165 | 0.175 |

| Average Value | 0.068 | 0.066 | 0.070 | 0.067 | 0.064 | 0.072 |

| Minimum Value | 0.007 | 0.006 | 0.008 | 0.007 | 0.005 | 0.009 |

|---------------------|---------|---------|---------|---------|---------|---------|

Fig **31 Table: Stress cloud for maximum driving force condition**

| Maximum Stress | 80.74 | 80.7 | 80.8 | 80.7 | 80.6 | 80.9 |

| Average Stress | 32.3 | 32.1 | 32.5 | 32.2 | 32.0 | 32.6 |

| Minimum Stress | 3.5 | 3.4 | 3.6 | 3.5 | 3.3 | 3.7 |

|---------------------|---------|---------|---------|---------|---------|---------|

Fig **32 Table: Emergency stop condition displacement map**

| Maximum Value | 0.060 | 0.058 | 0.062 | 0.059 | 0.055 | 0.065 |

| Average Value | 0.024 | 0.022 | 0.026 | 0.023 | 0.020 | 0.028 |

| Minimum Value | 0.003 | 0.002 | 0.004 | 0.003 | 0.001 | 0.005 |

|---------------------|---------|---------|---------|---------|---------|---------|

Fig **33 Table: Emergency stop condition stress map**

| Maximum Stress | 25.98 | 25.9 | 26.0 | 25.9 | 25.8 | 26.1 |

| Average Stress | 10.2 | 10.0 | 10.4 | 10.1 | 9.8 | 10.6 |

| Minimum Stress | 2.0 | 1.9 | 2.1 | 2.0 | 1.8 | 2.2 |

|---------------------|---------|---------|---------|---------|---------|---------|

Fig **34 Table: Displacement cloud at maximum lateral force condition**

| Maximum Value | 0.070 | 0.068 | 0.072 | 0.069 | 0.065 | 0.075 |

| Average Value | 0.028 | 0.026 | 0.030 | 0.027 | 0.024 | 0.032 |

| Minimum Value | 0.005 | 0.004 | 0.006 | 0.005 | 0.003 | 0.007 |

|---------------------|---------|---------|---------|---------|---------|---------|

Fig **35 Table: Stress map for maximum transverse force condition**

| Maximum Stress | 191.10 | 191.0 | 191.2 | 191.1 | 190.9 | 191.3 |

| Average Stress | 76.4 | 76.2 | 76.6 | 76.3 | 76.0 | 76.8 |

| Minimum Stress | 5.9 | 5.8 | 6.0 | 5.9 | 5.7 | 6.1 |

|---------------------|---------|---------|---------|---------|---------|---------|

Fig **36 Table: First six orders of modal shapes**

| 1st Order | 195.46 | 195.4 | 195.5 | 195.4 | 195.3 | 195.6 |

| 2nd Order | 195.47 | 195.4 | 195.5 | 195.4 | 195.3 | 195.6 |

| 3rd Order | 270.35 | 270.3 | 270.4 | 270.3 | 270.2 | 270.5 |

|---------------------|---------|---------|---------|---------|---------|---------|
